# Metabolic Alterations in Cardiomyocytes of Patients with Duchenne and Becker Muscular Dystrophies

**DOI:** 10.3390/jcm8122151

**Published:** 2019-12-05

**Authors:** Gabriella Esposito, Antonella Carsana

**Affiliations:** 1 Department of Molecular Medicine and Medical Biotechnologies, University of Naples Federico II, Via Pansini 5, 80131 Naples, Italy; gabriella.esposito@unina.it; 2 CEINGE Advanced Biotechnologies, Via Gaetano Salvatore 486, 80145 Naples, Italy

**Keywords:** cardiomyopathy, Duchenne and Becker muscular dystrophy, metabolic alterations, mitochondrial dysfunction

## Abstract

Duchenne and Becker muscular dystrophies (DMD/BMD) result in progressive weakness of skeletal and cardiac muscles due to the deficiency of functional dystrophin. Respiratory failure is a leading cause of mortality in DMD patients; however, improved management of the respiratory symptoms have increased patients’ life expectancy, thereby also increasing the clinical relevance of heart disease. In fact, the prevalence of cardiomyopathy, which significantly contributes to mortality in DMD patients, increases with age and disease progression, so that over 95% of adult patients has cardiomyopathy signs. We here review the current literature featuring the metabolic alterations observed in the dystrophic heart of the *mdx* mouse, i.e., the best-studied animal model of the disease, and discuss their pathophysiological role in the DMD heart. It is well assessed that dystrophin deficiency is associated with pathological alterations of lipid metabolism, intracellular calcium levels, neuronal nitric oxide (NO) synthase localization, and NO and reactive oxygen species production. These metabolic stressors contribute to impair the function of the cardiac mitochondrial bulk, which has a relevant pathophysiological role in the development of cardiomyopathy. In fact, mitochondrial dysfunction becomes more severe as the dystrophic process progresses, thereby indicating it may be both the cause and the consequence of the dystrophic process in the DMD heart.

## 1. Introduction

**Figure 1:**
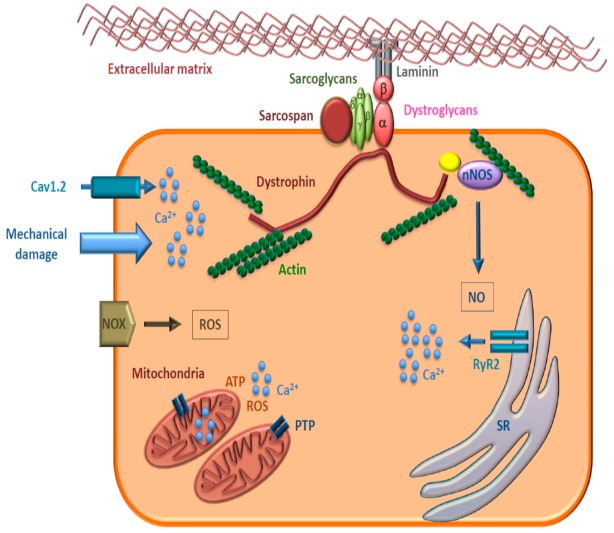
Schematic representation of the dystrophin-associated glycoprotein complex in cardiomyocytes. Dystroglycans, sarcoglycans, and other key proteins involved are shown. nNOS: neuronal nitric oxide synthase, NO: nitric oxide, Cav1.2: cardiac voltage-dependent L-type calcium channel, NOX: NADPH oxidase, ROS: reactive oxygen species, PTP: permeability transition pore, RyR2: ryanodine receptor type 2, SR: sarcoplasmic reticulum.

Duchenne (DMD, OMIM #301200) and Becker muscular dystrophies (BMD, OMIM #300376) are X-linked recessive degenerative disorders caused by mutations in the dystrophin gene (*DMD*, HGNC:2928). Dystrophin (DMD, OMIM *300377) is a part of the dystrophin-associated glycoprotein complex (DGC) that connects the cytoskeleton to the extracellular matrix [1]. Dystrophin binds to actin filaments in the cytoskeleton and to DGC proteins in the plasma membrane and is essential for the sarcolemmal structure and for protection from mechanical stress [2]. DGC also interacts with proteins implicated in signaling pathways, such as neuronal nitric oxide synthase (nNOS or NOS1), phosphoinositol triphosphate 2, calmodulin, and growth factor receptor-bound protein 2 [3]. Moreover, DGC is involved in the extracellular signal-regulated kinases (ERK)/mitogen-activated protein kinase (MAPK) signaling cascade [4] and is required for the clustering of ion channels and Ca^2+^ homeostasis [5]. The loss of functional dystrophin disrupts the DGC and NOS1, causing a decrease in sarcolemmal structural integrity, as well as susceptibility to myofiber injury and dystrophy development (Figure 1).

The incidence of DMD and BMD is approximately 1 in 3500 and 1 in 20,000–30,000 live male births, respectively [6]. DMD and, often, BMD are lethal conditions. Effective treatments are limited for DMD patients, and research for genetic-based therapies is ongoing [7]. As a consequence, the analysis of the *DMD* gene is of utmost importance for the identification of the underlying molecular defect, because it can confirm the clinical diagnosis, reveal patients’ genotype, address patients to the most opportune therapeutic options, and allow the identification of carrier females and the application of prenatal tests [8,9,10].

Patients affected by DMD lack the dystrophin protein and show progressive degeneration of skeletal muscles at 3–5 years of age and inability to walk at the age of about 10–12 years; their average life expectancy is of about 30 years of age. BMD patients have reduced content of the dystrophin protein and show a broad spectrum of clinical symptoms, a later disease onset, and a slower progression, with difficulties in ambulation that appear at a median age of 20 years [6]. The cardiomyocytes of DMD patients exhibit susceptibility to mechanical stress that contributes to heart fibrosis [11] and to the development of the often-lethal dilated cardiomyopathy (DCM) [12,13]. The cardiac pathology and the altered respiratory function caused by diaphragm damage leading to DCM are present in almost all DMD patients over 30 years of age and are the major causes of death at about 40 years of age [14]. In patients with mild forms of BMD, symptoms are evident at the age of 30, and patients may be still ambulant at 60 years of age; however, they experience worse cardiomyopathy than DMD patients, and about 70% of them have left ventricular dysfunction [14]. DMD/BMD carrier females are usually asymptomatic, although some of them have clinical symptoms, due to X-chromosome rearrangements involving the dystrophin locus or to unbalanced X inactivation [15].

Sarcolemmal fragility due to the absence of dystrophin is associated with elevated cellular Ca^2+^ that causes altered cell signaling, necrosis of myofibrils, fibrosis, inflammation, and vascular dysfunction in DMD patients. Therefore, dystrophinopathies are systemic diseases involving skeletal and cardiac muscle myopathy, chronic inflammation, impaired signaling and metabolism. Mouse models of DMD presenting disease progression similar to that in humans have been developed [16,17]. Recently, the hypothesis has been advanced that DMD/BMD are primarily metabolic diseases, and the sarcolemmal damage is a downstream sequela [18].

The aim of this paper is to review the main metabolic alterations occurring in the cardiomyocytes of DMD patients.

## 2. Materials and Methods

**Figure 2:**
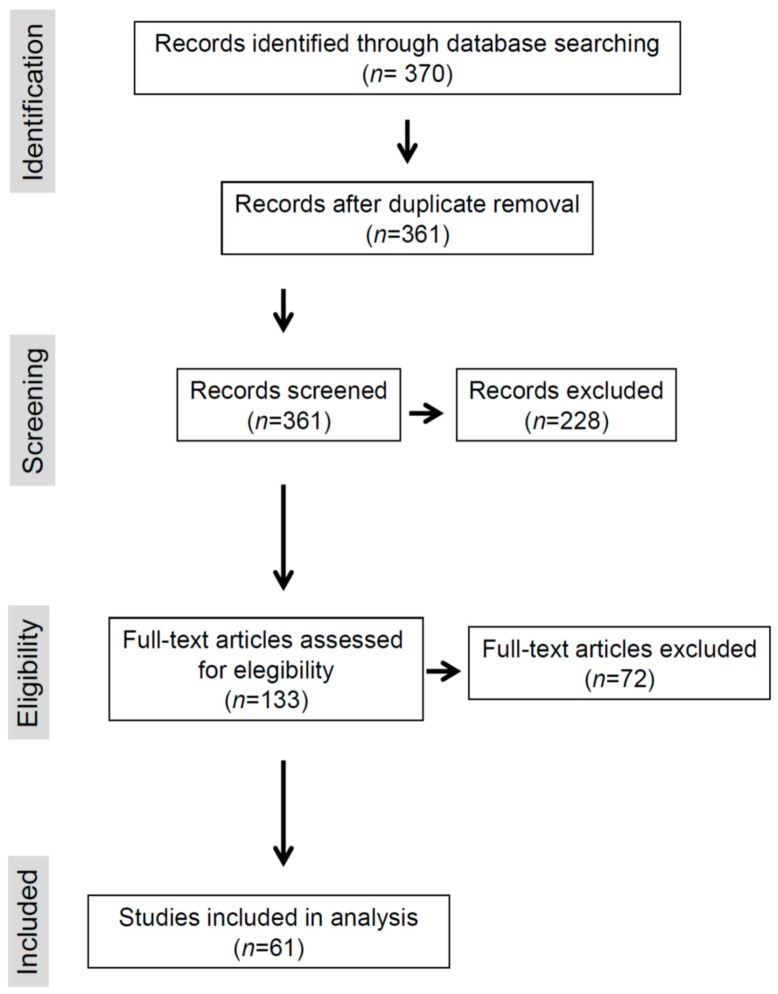
Preferred Reporting Items for Systematic Reviews and Meta-Analysis (PRISMA) flow chart.

This review was conducted according to the Preferred Reporting Items for Systematic Reviews and Meta-Analyses (PRISMA) methodology [19]. The search covered the PubMed database (Figure 2). The publications’ search period ranged from 2008 to 2019. The search term used was “Duchenne Becker” combined with “metabolism”, “cardiomyopathy, metabolism”, or “cardiovascular cardiomyopathy metabolism”. The selection of papers was based first on the titles and then on the abstracts. A word file containing the selected papers was developed and available to all the co-authors. To be included in the analysis, studies had to report alterations of cardiac metabolism in DMD/BMD patients or in animal models. Full texts of selected articles were then analyzed, and only papers that reported and discussed results supported by stringent experimental data carried out with appropriate methodologies were included. Any issue encountered by an author when extracting the data was discussed collectively, and a consensus was adopted to harmonize the extraction process. Manual searches were also made using reference lists from the recovered articles. In total, 51 references found by manual search were included; they concerned either articles reporting alterations of cardiac metabolism in DMD/BMD patients or in animal models published before 2008 or articles dealing with general matter related to the topic. Figure 2 describes the flowchart of this process.

## 3. Results

### 3.1. Lipid Metabolism

Alterations in plasma and tissue lipids have been reported in DMD patients [20], and it has been proposed that plasma lipids significantly contribute to pathology and that DMD patients could benefit from lipid-lowering and vascular targeted therapies [21] (Table 1). Interestingly, it has been reported that statins are pleiotropic drugs, and, in addition to their effect on lowering cholesterol, they are involved in the regulation of processes implicated in DMD progression, such as autophagy and NADPH oxidase 2-mediated oxidative stress (see also Section 3.3 *Reactive Oxygen/Nitrogen Species (ROS/RNS)*) [22], and angiogenesis [23].

Accumulation of phosphatidylcholine, cholesterol, sphingomyelin, triglyceride, and increase in monounsaturated fatty acid species have been detected in the muscles of DMD patients, while no major modification of lipid metabolism was observed in BMD patients’ muscles, except for reduced carnitine concentrations.

**Figure 3:**
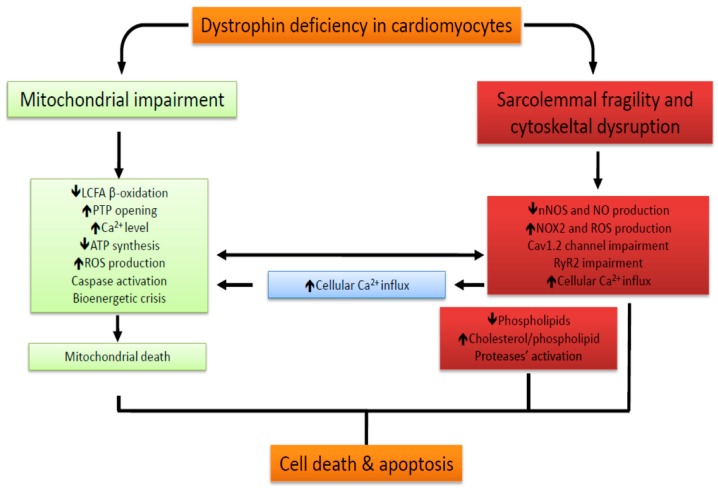
Metabolic alterations in Duchenne muscular dystrophy/Becker muscular dystrophy (DMD/BMD) cardiomyocytes. Dystrophin deficiency leads to sarcolemmal and cytoskeletal disruption and is associated with mitochondrial dysfunction. As a consequence, metabolic alterations, which are mainly represented by impaired Ca^2+^ homeostasis, oxidative stress, and bioenergetic impairment, occur both in the cytosol (red box) and in the mitochondria (in green) and lastly cause cell death and apoptosis.

In particular, the cardiac involvement in DMD was investigated using animal models of DMD with congestive cardiomyopathy [28,29]. In these animals, the total phospholipid content is significantly reduced only in the heart and not in skeletal muscles; however, the phosphatidylcholine amount and the cholesterol-to-phospholipid ratio are increased in both cardiac and skeletal muscles [30,31] (Figure 3). Moreover, lower activity and expression of fatty acid synthase and stearoyl-CoA desaturase in the liver, as well as decreased insulin levels have been detected in DMD mice compared to control animals [32]. Insulin deficiency may in part cause impaired fatty acid metabolism. The increase in cholesterol-to-phospholipid ratio could be ascribed to the release of phospholipids during muscle degeneration, to increased activity of phospholipase A, and to increased cholesterol synthesis during muscle regeneration [33].

### 3.2. Mitochondrial Impairment/Dysfunction

Cardiomyocytes are cells with elevated energy requirements that therefore are highly dependent on mitochondrion activity. In these cells, mitochondria occupy approximately one-third of the cell volume, actively synthesize ATP by oxidative phosphorylation, and are the major source of reactive oxygen species (ROS) that can trigger oxidative stress and affect cell survival and death [34]. Moreover, mitochondria are site for Ca^2+^ storage, because they release and withdraw Ca^2+^ to and from the cell [35].

Dystrophin deficiency disrupts sarcolemmal stability and cytoskeletal organization, thereby triggering a variety of cellular stress factors. Among these, increased oxidative stress, impaired handling of cellular Ca^2+^, and strong decrease in nitric oxide (NO) signaling due to an impaired activity of nNOS (or NOS1, OMIM *163731) have been reported in DMD patients and in a dystrophin-deficient mouse model of DMD (*mdx* mouse) [36]. Moreover, in the initial compensatory phase that anticipates clinical heart manifestations, cardiac remodeling shifts the energy production from mitochondrial β-oxidation of long-chain fatty acids, which provides about three-quarters of the heart’s energy requirement, to extra-mitochondrial oxidation of carbohydrates [37]. All these findings indicate that mitochondrial metabolic alterations are present in DMD hearts before cardiomyopathy becomes overt.

Another prominent early factor in cardiomyopathy progression in DMD is the increased susceptibility of mitochondria to open the permeability transition pore (PTP), a cyclosporine A- sensitive high-conductance channel in the inner mitochondrial membrane (IMM) (Figure 1). As consequence of PTP opening, which plays a key role in the pathogenesis of diseases due to necrotic cell death after ischemic injuries or to muscle and brain degeneration [38], a nonspecific channel with an exclusion size of 1.5 kDa is formed within the IMM. Physiological transient PTP opening allows a rapid Ca^2+^ release and metabolite exchange between mitochondrial matrix and cytosol; in contrast, persistent opening leads to pathological wasting of the IMM potential, resting of ATP synthesis, bioenergetic crisis, and cell death—a main feature of mitochondrial disorders [38]. In the hearts of young *mdx* mice, before any clinical evidence of cardiomyopathy, mitochondria that undergo PTP opening are significantly more numerous than in normal hearts, thereby indicating that PTP opening has a key role in the pathogenesis of dystrophic cardiomyopathy (Figure 3). Accumulation of Ca^2+^ in the mitochondrial matrix of dystrophic muscle cells is one the main triggers of permeability transition [38]. In these cells, Ca^2+^ levels can increase as a consequence of the lack of dystrophin that favors sarcolemma disruption during mechanical stress and, in the absence of sarcolemma disruption, through the voltage-independent stretch-sensitive Ca^2+^ leak channels, the store-operated Ca^2+^ channels, and the ryanodine receptor [37].

Also, the increase in ROS production may effectively induce PTP opening [38]. A major source of ROS production in the muscle is the NADPH oxidase (NOX) enzyme. Expression and activity of the NOX2 isoform are increased in cardiac myocytes from *mdx* mice (see also Section 3.3 *Reactive Oxygen/Nitrogen Species (ROS/RNS)*) [39]. The activation of NOX2 produces extracellularly the superoxide anion (O_2_^•−^), which is converted to membrane-permeant H_2_O_2_ by extracellular superoxide dismutase [39,40]. High levels of ROS lead to apoptosis or necrosis [34]. Oxidative stress that is present in mitochondria from dystrophic hearts, increased PTP opening, and activation of caspase 9/3 reported in young *mdx* hearts before the onset of cardiac impairment are all suggestive of mitochondria-derived apoptosis [41] (Figure 3).

A very recent study demonstrates that, during altered oxidative phosphorylation, complex I-sustained emission of mitochondrial H_2_O_2_ increases in the left ventricle of dystrophin-deficient young mice, before any evidence of cardiac dysfunction [42]. Therefore, the identification of early mitochondria-specific impairments may lead to the development of mitochondria-targeted therapies able to recover respiratory chain activity and bioenergetic control, with the aim to delay the onset of cardiomyopathy and the consequent progression to heart failure in DMD.

### 3.3. Reactive Oxygen/Nitrogen Species (ROS/RNS)

ROS-induced oxidative stress contributes to damage in Ca^2+^ handling and correlates with cardiomyopathy progression and the severity of heart failure in DMD patients. Superoxide anion (O_2_^•−^) is the main free radical formed in muscles.

The membrane-bound enzyme NOX is the major source of O_2_^•−^ in the cardiovascular system (Figure 1). Increased expression of the isoform NOX2 and O_2_^•−^ production have been reported in the hearts of *mdx* mice compared to those of wild-type controls [43]. NOX2 inhibition determines the reduction of ROS levels toward levels similar to those of wild-type mice and restores the sarcoplasmic reticulum (SR) Ca^2+^ content and the amplitude of evoked intracellular Ca^2+^ concentration transients that are decreased in *mdx* mice [39,43]. The impairment of Ca^2+^ handling by oxidative stress occurs essentially at the level of the RyR2 and Cav1.2 calcium channels, which are redox-sensitive (see also Section 3.4 *Calcium Handling*). Consequently, increased ROS levels may in part explain the Ca^2+^ channel abnormalities in dystrophic cardiomyocytes [36,44] (Figure 3 and Table 1).

The RNS NO is synthesized by NOS from L-arginine and oxygen and modulates a wide range of physiological functions. NOS1 is a member of the DGC and is lost in a situation of dystrophin deficiency [45]. Therefore, NOS1 deficiency could be the proximal cause of many of the poorly understood features of the dystrophic phenotype. In particular, NO protects the cardiac muscle through vascular relaxation and prevention of pathological hypertrophy [46]. However, there is no evidence indicating that dystrophin and NOS1 co-localize at the membrane of wild-type mouse cardiomyocytes [47]; on the contrary, NOS1 is localized at the intercalated discs, and its mislocalization is associated with DMD cardiomyopathy [48]. This implies that the physical proximity of dystrophin and NOS1 is not a key factor in NOS1 regulation in the heart and suggests an indirect, though efficient, role for dystrophin in modulating NOS1 activity. Selective pharmacological inhibition of NOS1 in wild-type cardiomyocytes lowers NO production, indicating that NOS1 is the main NOS isoform in cardiomyocytes [47]. Cardiomyocytes from *mdx* mice produce significantly lower NO levels than cardiomyocytes from wild-type mice (Figure 3) [47]. The use of transgenic NOS1 over-expression in *mdx* mice prevents the development of many signs of cardiomyopathy [49]. Therapeutic strategies have been developed to address neuronal nitric oxide synthase deficiency and the loss of nitric oxide availability in DMD [27] (Table 1).

### 3.4. Calcium Handling

Changes in excitation–contraction coupling and intracellular Ca^2+^ handling have been reported in the hearts of *mdx* mice.

The ryanodine receptor type 2 (RyR2, OMIM *180902) calcium channel and the voltage-dependent L-type calcium channel (Cav1.2, OMIM *114205) are the two principal channels involved in excitation–contraction coupling in the cardiac muscle. The activation of Cav1.2 by plasma membrane depolarization allows Ca^2+^ to flow into the cell; Ca^2+^ binds to RyR2, a large homotetrameric Ca^2+^ release channel expressed in cardiomyocytes and located on the SR membrane (Figure 1), and induces it to open and to release Ca^2+^ from the SR, thus triggering muscle contraction. This mechanism is called calcium-induced calcium release (CICR). Several proteins, and also ATP, cations, such as Ca^2+^, Mg^2+^, and pharmacological ligands regulate RyR2 activity.

Enhanced RyR2 activity has been associated with the pathogenesis of heart dysfunction in DMD [50,51,52]. RyR2 protein levels are two- to three-fold greater in the hearts of dystrophin-deficient *mdx* mice compared to those of wild-type mice [53]. Moreover, the increased protein kinase A (PKA)-mediated phosphorylation of RyR2 at serine 2808 (S2808) after β-adrenergic activation contributes to SR Ca^2+^ leak and to the development of heart failure [50]. In fact, inhibition of RyR2-S2808 phosphorylation in *mdx* mice largely prevents the development of age-related cardiomyopathy. Indeed, progressive cardiac impairment in *mdx* mice seems to depend on the synergistic contribution of both phosphorylation and oxidation of RyR2 [54]. Consistently, inhibition of RyR2 phosphorylation suppresses SR Ca^2+^ leak in the *mdx* mouse heart in part by reducing RyR2 oxidation [54]. Therefore, increased Ca^2+^ leak from the SR plays a crucial role in the development of cardiomyopathy in DMD. Despite the lower NO levels produced in the cardiomyocytes of *mdx* mice compared to those of wild-type mice [47], it has been reported that RyR2 is S-nitrosylated in *mdx* mice, resulting in a functional remodeling of the RyR2 complex and in the dissociation of RyR2 from its stabilizing subunit calstabin 2. These changes destabilize the RyR2 structure and increase SR Ca^2+^ leakage, leading to intracellular Ca^2+^ increase and a diastolic SR Ca^2+^ leak (Figure 3) [52]. This remodeling is analogous to the one observed in the skeletal muscle RyR1 channel complex following RyR1 S-nitrosylation [55]. The inhibition of calstabin 2 dissociation from the RyR2 complex suppresses the SR Ca^2+^ leak in cardiomyocytes and prevents arrhythmias in vivo. Then, rescue of the RyR2-mediated diastolic SR Ca^2+^ leak prevents fatal sudden arrhythmias in DMD hearts [52].

The voltage-dependent L-type calcium channel Cav1.2 co-localizes with dystrophin [56] and is linked to F-actin networks by subsarcolemmal stabilizing proteins that finely regulate the channel function [57]. Disruption of actin filaments significantly alters the Ca^2+^-L current [58]. Cardiomyocytes from adult *mdx* mice show enhanced Ca^2+^ current densities and impaired Ca^2+^- and voltage-dependent inactivation of the Cav1.2 channel compared to wild-type cardiomyocytes [59]. Ca^2+^ channel alterations in dystrophic cardiomyocytes seem to be dependent on mice age and become more severe in the adult age; in fact, a significantly reduced Ca^2+^ channel inactivation has been observed in the cardiomyocytes of neonatal *mdx* mice [60]. Therefore, enhanced Ca^2+^ influx through Cav1.2 may contribute to cardiomyopathy development (Figure 3) [60]. However, the functional properties of Cav1.2 are similar in the cardiomyocytes from aged (>1 year of age) *mdx* and wild-type mice [61]. The loss of Cav1.2 dysregulation in the heart during aging can be ascribed to the significant decrease of dystrophin protein in the senescent murine heart. In fact, the altered L-type Ca^2+^ currents in dystrophic hearts have been explained by an impaired Cav1.2 regulation in the absence of dystrophin. Moreover, increased basal phosphorylation of the Cav1.2 alpha1C subunit and enhanced PKA activity after β-adrenergic activation in the hearts of *mdx* mice have been reported [62]. PKA-mediated phosphorylation of Cav1.2 enhances the L-type Ca^2+^ currents and also affects the channel inactivation properties [63]. The enhanced PKA activity in dystrophic cardiomyocytes also explains the enhanced PKA-mediated phosphorylation of RyR2 associated with dystrophic cardiomyopathy in *mdx* mice.

Another potential source of L-type Ca^2+^ currents alterations in dystrophic cardiomyocytes is the redox modification of the Cav1.2 alpha1 subunit (cysteine 543 oxidation) during oxidative stress [64], which results in an increase in the channel-mediated Ca^2+^ influx [63].

Although it is well accepted that Cav1.2 is inhibited by NO via NOS1 activity in the heart, some recent data do not confirm or even contradict this view [65]; therefore, the role of NO and NOS activity in regulating Cav1,2 in the heart is still debated [65].

Sarco/endoplasmic reticulum Ca^2+^-ATPase (SERCA) pumps Ca^2+^ from the cytosol into the lumen of the SR, using the energy derived from ATP hydrolysis and is essential for the maintenance of a low cytosolic Ca^2+^ concentration. SERCA2 protein expression in *mdx* mice is not significantly different compared to that in wild-type hearts [53]. However, SERCA2 is reversibly inhibited by phospholamban (PLN); phosphorylation of PLN at serine 16 and threonine 17 reverses this inhibition. The phosphorylated PLN monomer content is lower in *mdx* compared to wild-type hearts. This suggests that SERCA2 activity is affected in *mdx* mice, leading to increased decay constants of Ca^2+^ transients [53].

Intracellular calcium overload in DMD cardiomyocytes can also arise form an altered function of other channels, such as Na^+^–H^+^ exchanger (NHE-1) and proton channels [66]. An increased Na^+^ influx through NHE-1 leads to an intracellular accumulation that, in turn, promotes calcium influx through the Na^+^–Ca^+2^ exchanger.

## 4. Discussion

Metabolic impairment is evident not only in skeletal muscle but also in many tissues and cells from DMD patients and animal models, including heart, liver, and brain. Therefore, it has been proposed that DMD is characterized by a systemic metabolic impairment (Figure 3)—which is central to the etiology of the disease and not secondary to its pathophysiology—and is primarily a mitochondrial myopathy [18]. Disruption of sarcolemmal membrane and cytoskeletal organization are associated with several cellular alterations, i.e., elevated cytosolic Ca^2+^, oxidative stress, and cell death, that may cause mitochondrial dysfunctions and ultimately contribute to muscle fiber degeneration. Already in 1992, Bonsett and Rudman [67] provided stringent evidence that adenylosuccinic acid (ASA) treatment can induce positive effects in DMD patients because it restores the mitochondrial metabolic impairment; indeed, ASA stimulates the Krebs and purine nucleotide cycles for ADP resynthesis, thereby increasing mitochondrial ATP synthesis. ASA treatment actually induces broad improvements in creatine retention and in the histology, energy levels, and strength of dystrophic muscles [67], which are partially lost if the ASA therapy is suspended.

Very recently, it has been demonstrated that mitochondrial impairment anticipates the onset of cardiomyopathy in a mouse model of DMD [42]. In particular, elevated mitochondrial H_2_O_2_ emission and impaired oxidative phosphorylation have been detected in the left ventricle muscle of these mice at an early age, when signs of cardiac dysfunction are absent, suggesting that mitochondrial dysfunction plays a role in the etiology of the heart disease occurring at an older age [42].

Although changes induced by dystrophin deficiency, i.e., oxidative stress and impairment of Ca^2+^ homeostasis, are involved in the development of cardiomyopathy in DMD patients, mitochondrial dysfunction potentially contributes to cardiac dysfunction. Indeed, treatment with idebenone—a quinone-based electron shuttle—improved cardiac and respiratory performance in DMD patients [24,25,26] (Table 1), thereby providing further proof that the mitochondria are implicated in DMD-associated heart dysfunction. Therefore, considering and treating DMD as a metabolic disease could improve DMD therapies and the quality of life of patients.

## Figures and Tables

**Table 1:** Metabolic targets to treat cardiomyopathy in DMD/BMD patients.

Dysfunctional Metabolism	Molecular Alteration	Therapeutic Target	Available Drugs	Potential Therapeutic Strategy
**Lipids**	Increased cholesterol-to-phospholipid ratio	Cholesterol synthesis	Statin	
**Mitochondria**	Increased O_2_^•−^ production			
	Impaired Ca^2+^ handling			
	Impaired oxidative phosphorylation	Respiratory complex I function	Idebenone [24,25,26]	
**ROS**	Increased expression of NOX2 Increased O_2_^•−^ production	NOX2	Statin [22]	NOX2 inhibition
**RNS**	Lower NO levels Impaired NOS1 activity	NO delivery NO synthesis	NO donors [27]	
